# Green Synthesis of Chitosan-Coated Selenium Nanoparticles for Paclitaxel Delivery

**DOI:** 10.3390/nano15161276

**Published:** 2025-08-18

**Authors:** Mouhaned Y. Al-Darwesh, Maroua Manai, Hammouda Chebbi, Axel Klein

**Affiliations:** 1 Laboratory of Materials, Crystal Chemistry and Applied Thermodynamics, Faculty of Sciences of Tunis, University of Tunis El Manar, El Manar II, Tunis 2092, Tunisia; chebhamouda@yahoo.fr; 2 Department of Chemistry, College of Science, University of Anbar, Ramadi 31001, Iraq; 3 Laboratory of Transmission, Control and Immunobiology of Infections, Pasteur Institute of Tunis, University of Tunis El Manar, 13 Place Pasteur, 1002 Tunis le Belvedere, Tunis 2092, Tunisia; marwa.manai@pasteur.utm.tn; 4 Preparatory Institute for Engineering Studies of Tunis, University of Tunis, Jawaharlal Nehru Street, 1089, Montfleury, Tunis 2092, Tunisia; 5 Institute for Inorganic and Materials Chemistry, Department of Chemistry and Biochemistry, Faculty of Mathematics and Natural Sciences, University of Cologne, Greinstrasse 6, 50939 Köln, Germany

**Keywords:** *F. vulgare* seed extract, selenium nanoparticles, green synthesis, paclitaxel, antiproliferative activities

## Abstract

Selenium nanoparticles (Se NPs) were synthesized from Na_2_SeO_3_ using *Foeniculum vulgare* (fennel) seed extract as mild sustainable reductant, coated with chitosan (Ch), and loaded with Paclitaxel (PTX). The PTX release from the Se@Ch–PTX NPs and their cytotoxicity against MDA-MB-231 breast cancer cells was studied in view of an application as drug delivery platform. Thermogravimetric analysis (TGA) showed the thermal stability of the NPs up to 300 °C. UV–vis absorption and Fourier transform IR (FT-IR) spectroscopy allowed to trace surface species originating from the *F. vulgare* extract on the Se NPs, while the surface of the Se@Ch–PTX NPs is characterized from Ch and PTX functionalities. Scanning electron microscopy (SEM) and transmission electron microscopy (TEM) showed approximate spherical shaped NPs with sizes ranging from 10 to 40 nm. Zeta potential measurements showed a clear distinction between the −39 mV found the Se NPs and +57 mV for the Ch–PTX coated NPs. The NPs showed good biocompatibility with red blood cells (RBCs) in hemolytic activity assays, exhibiting no hemolytic effects at concentrations ranging from 50 to 400 µg/mL. In vitro release studies showed a sustained and pH-responsive release pattern with a maximum release of about 80% within 22 h for Se@Ch–PTX at pH = 3.5. The Se@Ch–PTX NPs showed high antiproliferative activity against MDA-MB-231 cells with an IC_50_ value of 12.3 µg/mL compared to about 36 for PTX and 52 µg/mL for the Se NPs. The reactive oxygen species (ROS) activity as studied through DPPH scavenging showed higher values for the Se@Ch–PTX NPs compared to the Se NP.

## 1. Introduction

Breast cancer is the most common type of cancer among women worldwide, posing a significant public health threat with millions of cases annually [1,2,3]. Established treatments, including surgery, radiotherapy, and chemotherapy, face significant challenges, including severe side effects, limited selectivity in targeting cancer cells, and the development of drug resistance [4,5].

Paclitaxel (PTX) is a very prominent chemotherapeutic agent used in breast cancer treatment [6,7,8,9,10]. Initially discovered in the bark of the Pacific yew tree, PTX is known for its high cytotoxicity against cancer cells [9]. However, its use is associated with serious side effects, including neurotoxicity, poor bioavailability, and adverse effects on healthy cells, which limit its therapeutic efficacy [11,12]. Consequently, enhancing the efficiency of PTX and thus minimizing side effects through reduced doses is an important objective in cancer therapy research [13,14].

Nanoparticles (NPs) represent an innovative and promising approach as drug-delivery systems in cancer treatment, as NP can be functionalized to enhance solubility and bioavailability, and even allow the targeting of cancer cells [15,16,17,18,19]. Recent studies have shown that selenium NPs (Se NPs) are not only good candidates for NP-based drug delivery [19,20,21,22,23,24,25,26,27,28,29,30], but that Se additionally possesses anticancer properties by promoting oxidative stress in cancer cells through the production of reactive oxygen species (ROS) and thus inducing apoptosis [20,22,23,24]. Additionally, Se NPs are biocompatible and exhibit relatively low toxicity toward healthy cells, making them good candidates for cancer therapy [23,24].

A number of studies has thus used Se NPs for PTX delivery [25,26,27,28,29,30,31]. In a recent study, the nanocomposite Se@β-CD–FA–PTX was produced from Se NPs, β-cyclodextrin (β-CD) and folic acid (FA), using a layer-by-layer assembly method, and loaded with PTX. This system is capable of entering cancer cells through folate receptor-mediated endocytosis, enabling targeted intracellular drug delivery [27]. In another study, Se NPs conjugated with hyaluronic acid and loaded with PTX (Se@HA–PTX) were studied and showed antiproliferative activities against A549 lung cancer cells by enhancing cellular uptake through HA receptor-mediated mechanisms [28]. Furthermore, Se NPs were loaded with PTX using Pluronic F-127 as a stabilizer and their anticancer activity was evaluated against various cancer cell lines (A549, MCF-7, HeLa, HT29) [29]. The induction of G2/M phase cell cycle arrest and the promotion of apoptosis via mechanisms involving mitochondrial membrane potential disruption increased reactive oxygen species (ROS) production, and caspase activation was studied and discussed as possible mode of action [29].

In a number of reports, Se NPs for various purposes were produced from SeO_2_ or selenite using biological material as mild sustainable reductant [23,26,29,30,31,32,33,34,35,36,37,38,39,40,41,42]. FT-IR and UV–vis absorption spectroscopy of thus produced Se NPs clearly show that the reducing biomaterials have left a plethora of functional groups on the Se surface as protecting ligands. These surface ligands probably contribute also to the biological activities of such Se NPs [30,31,32,33,34,35,37,42].

On the other hand, an important strategy in NP-based drug delivery is the use of defined polymeric materials that allow blending with drug molecules and slow, controlled release of drug molecules through slow degradation of the surface polymer [43]. Chitosan, a natural polymer derived from chitin, is known for its biocompatibility and non-toxic properties [44,45,46]. Its positive charge facilitates strong interactions with negatively charged cancer cell membranes, potentially enhancing cellular uptake [46,47,48].

**Scheme 1:**
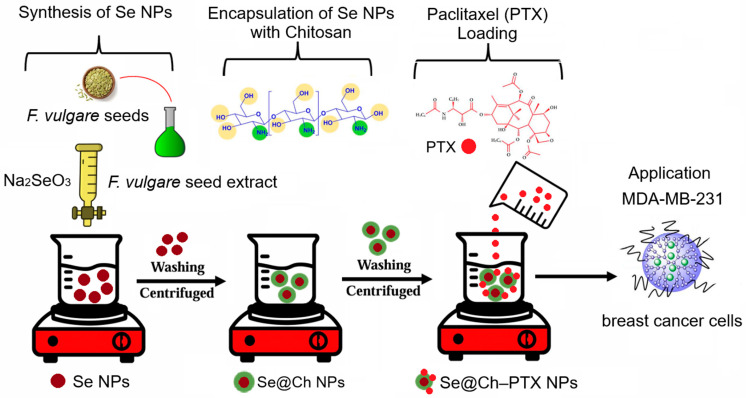
Schematic of the synthesis of Se@Ch–PTX NPs using *F. vulgare* seed extract.

Herein we report on the develop of a PTX drug-delivery system based on chitosan-covered Se NP, Se@Ch–PTX. We used the aqueous extract of *Foeniculum vulgare* (fennel) seeds to produce Se NPs, covered them with chitosan and loaded them with PTX (Scheme 1). We studied the PTX-release, the antiproliferative activity against MDA-MB-231 breast cancer cells, and the ROS activity. *F. vulgare* seed extract was chosen as mild reductant. Fennel represents as important medicinal plant widely used in traditional medicine for its therapeutic benefits [49,50,51,52,53,54]. It contains bioactive compounds such as anethole, fenchone, and estragole, which showed antioxidant, anti-inflammatory, and antibacterial properties [51,52]. Given its antioxidant properties, *F. vulgare* extract should be suitable as bio-reductant for the production of Se NPs and at the same time provide bio-active surface ligands [52,54].

## 2. Results and Discussion

### 2.1. Synthesis

Red Se NPs were synthesized from Na_2_SeO_3_ and *F. vulgare* seed extract as mild and sustainable (green) reductant at 30 °C in 60 min with a yield of 87%. The NPs were stabilized using phosphate-buffered saline (PBS) solution at pH = 7.4. The chitosan-coated NPs (Se@Ch) were synthesized by adding a chitosan glacial acetic acid mixture to the Se NPs. Paclitaxel (PTX) was loaded to the Se@Ch NP by stirring them in a PTX DMSO solution. The Se@Ch–PTX NPs were produced in 0.8 g quantity and can be stored in de-aerated PBS solution (pH = 7.4) under the exclusion of light for at least one week.

### 2.2. Analytic and Spectroscopic Characterization

The FT-IR spectrum of the *F. vulgare* extract (Figure S1 in the Supplementary Materials) shows characteristic bands at 3450, 2963, 1626, and 1123 cm^−1^ that have been previously reported for such materials [55]. The FT-IR of the Se NPs are quite similar to those of the extract, in keeping with our intention that characteristic components of the extract cover the Se NPs as stabilizing ligands. In the FT-IR spectrum of the Se@Ch–PTX, a marked shift of the broad band centered around 3400 cm^−1^ and a broad band at around 600 cm^−1^ point to the presence of Ch and PTX on the particles. Unfortunately, the spectrum does provide any information about the way PTX is loaded onto the Se@Ch NPs and if *F. vulgare* components are still present in the Ch–PTX shell around the Se NPs.

In the UV–vis absorption spectrum (Figure S2) the *F. vulgare* extract shows a broad band peaking at 245 nm. The Se NPs are characterized by a maximum at 280 nm shoulders at 360, 450, and 600 nm, in keeping with previous reports [56,57,58]. For the Se@Ch–PTX NPs three distinct absorptions were recorded at 269, 355, and 448 nm, which represents a slight variation in the Se NP absorption and is probably due to the Ch–PTX functionalization [59,60].

The powder X-ray diffractogram of the Se@Ch–PTX NPs (Figure S3) shows the characteristic signals of highly crystalline Se in its hexagonal structure, in line with previous reports [39,40,61] and in agreement with the standard [61]. Using the Debye–Scherrer equation, the nanoparticle size of the synthesized particles was calculated to 33.6 nm. Additional peaks indicate the presence of PTX and chitosan in the coating, but also do not provide any information on the binding of PTX and Ch on the Se surface.

The Thermogravimetric Analysis (TGA) of the Se@Ch–PTX NPs shows a subtle weight loss up to 300 °C indicating loss of water molecules attached to the surface (Figure S4). From 300 to 420 °C the NPs underwent a loss of more than 50% of their weight, leaving a material that is stable up to 640 °C and then loses further 30% weight up to 750 °C, leaving a residual of 11% of the original mass.

The Zeta potential of the Se NPs was measured to −38.29 mV (Figure S5), in line with the presence of weakly bound surface ligands from the extract. Functionalization with chitosan and PTX led to a Zeta potential of +56.66 mV, which is probably due to the surface of the particles being completely covered by chitosan and PTX. The same behavior has previously been reported for Se@β-CD–FA@PTX (+30 mV) and Se@β-CD–FA NPs (+37 mV; FA = folate, β-CD = β-cyclodextrin), in contrast to pristine Se NPs (−25 mV) [27].

### 2.3. Scanning Electron (SEM) and Transmission Electron (TEM) Microscopy

**Figure 1:**
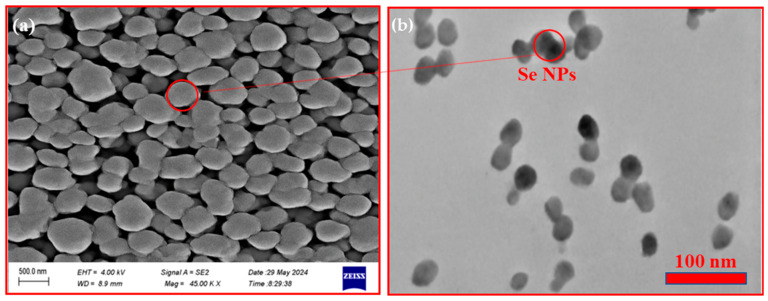
SEM (**a**) and TEM (**b**) of the Se NPs.

The SEM of the Se NPs (Figure 1a) showed approximately spherical shape NPs separated with clear boundaries, a rough granular surface, and an average size of 25.4 ± 9.3 nm. The TEM (Figure 1b) confirmed the spherical shape of the nanoparticles, with particle sizes ranging from 10 to 40 nm.

**Figure 2:**
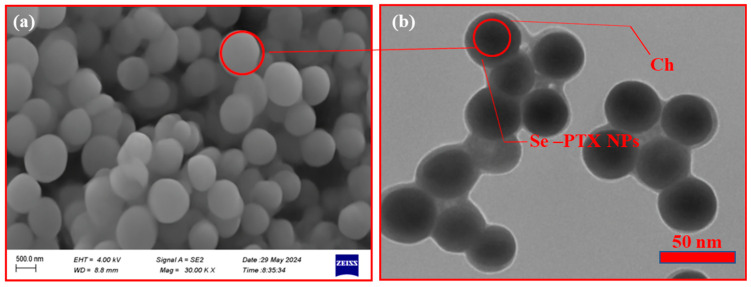
SEM (**a**) and TEM (**b**) of the Se@Ch–PTX NPs.

For the Se@Ch–PTX NPs, a far more pronounced spherical morphology was observed in the SEM (Figure 2a), with a markedly smaller size distribution ranging from 20 to 30 nm. The TEM (Figure 2b) confirms their spherical shape and size distribution. The transparent layer surrounding the particles represents likely the chitosan/PTX covering shell, confirming the complete coverage of the Se NP concluded from the Zeta potential measurements.

The analysis of the NP materials show that chitosan effectively coated the surface of the Se NPs, resulting in the formation of a stable core@shell structure as reported before [62]. Furthermore, chitosan contributes to nanoparticle stabilization against agglomeration through its highly positive Zeta potential of +56.66 mV. This observation aligns with previous reports demonstrating that chitosan significantly enhances the colloidal stability of metal-based nanoparticles through surface charge modification and steric protection [63,64].

### 2.4. In Vitro Drug Release

**Figure 3:**
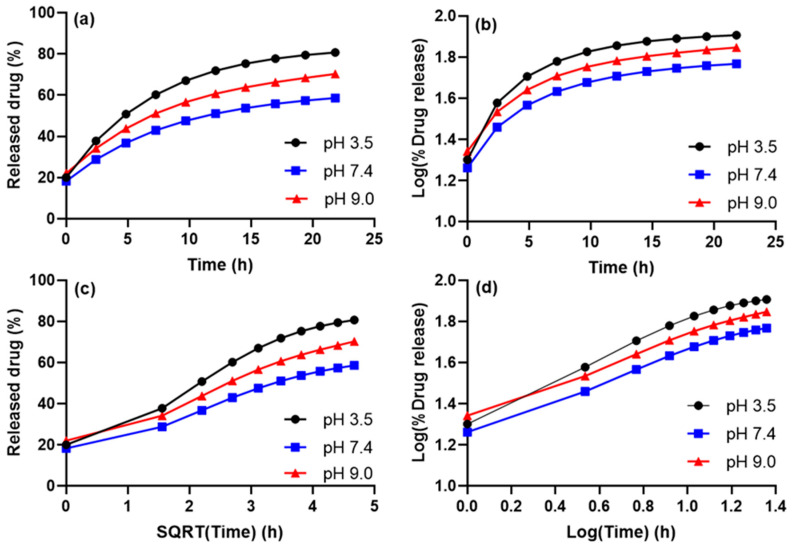
Kinetic models for release of PTX from Se@Ch–PTX NPs: (**a**) zero-order; (**b**) first order; (**c**) Higuchi; and (**d**) Korsmeyer–Peppas model of kinetics.

A rather controlled release of up to 80% PTX from Se@Ch–PTX NPs was found within 22 h at pH = 3.5 (Figure 3a). At higher pH = 7.4, and 9.0, the release was slower and lower of up to 62% at pH = 7.4 and 57% at pH = 9.

The release kinetics showed high conformity with the Korsmeyer–Peppas model (Figure 3d), with correlation coefficients *R*^2^ exceeding 0.98 (Table 1). This model is characteristic of drug release through a combined mechanism involving both diffusion and degradation of the biopolymer coating [43,53,65]. The agreement to the Higuchi model (Figure 3c) is only slightly lower. This model is applicable if diffusion plays the primary role [37]. In contrast to this, the zero-order release model (Figure 1a) showed quite moderate suitability, with *R*^2^ values ranging between 0.85 and 0.9, reflecting good control over the release rate over time and sustained drug release and also indicates system stability [38]. The first-order release model (Figure 1b) gave the poorest fit, confirming that drug release is influenced not only by the remaining drug concentration but also by factors such as surface composition and coating nature [66].

The release rates of our materials are markedly lower compared to those of very related Se@Ch–PTX NPs, for which a release of about 80% after 55 h at pH 5.5 and about 15% after 55 h at pH 7.4 was reported [26]. The reason for the higher release lies very probably in the much larger size of these NPs of about 140 nm, compared with the approximately 30 nm of our NPs. An interesting comparison was drawn for C-peptide-conjugated solid lipid nanoparticles as carriers for PTX [67]. While the C-peptide–SLN–PTX NPs showed controlled release of about 80% after 60 h at pH 5.5, the SLM-PTX material delivered 80% in 50 h, and PTX NPs within 5 h. These two studies and others show a strong dependence of the release on pH [26,27,28,67], thus confirming our findings.

### 2.5. Hemolysis Test

**Figure 4:**
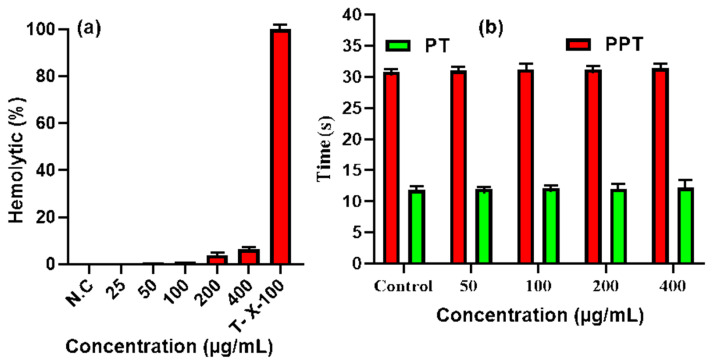
(**a**) Hemolytic activity of the Se@Ch–PTX NPs (**a**) compared with Triton-X-100. (**b**) Results of the PT (green bars) and aPPT assays (red bars) on Se@Ch–PTX NPs. Control for PT and aPPT = saline.

Treatment of red blood cells (RBCs) with the Se@Ch–PTX NPs showed only marginal hemolysis at 400 µg/mL (Figure 4a).

In contrast to this, a 100 µg/mL concentration of Triton-X-100 resulted in 100% hemolysis, which agrees with the membrane-disrupting nature of this detergent. Furthermore, aPTT (activated partial thromboplastin time) and PT (prothrombin time) assays showed that concentrations of Se@Ch–PTX NPs, ranging from 50 to 400 µg/mL did not cause any significant change (Figure 4b). The coagulation times remained within the normal physiological ranges for both PT (9.4–12.5 s) and aPTT (25.1–36.5 s), according to a reference [68].

### 2.6. Antiproliferative Properties

**Figure 5:**
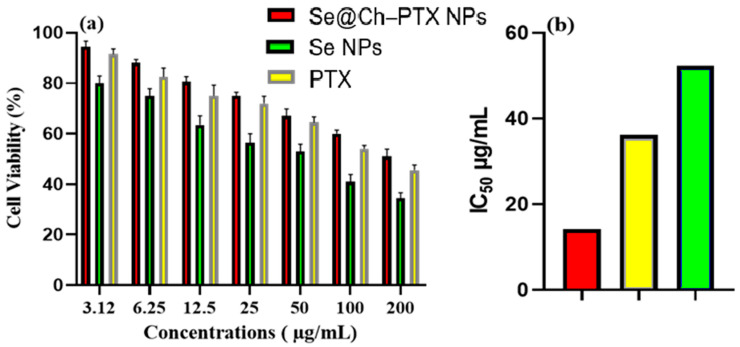
(**a**) Cell viability of MDA-MB-231 cells in the presence of PTX, Se NPs, and Se@Ch–PTX NPs at increasing concentrations. (**b**) IC_50_ values after 24 h treatment. All treatments were performed in triplicate. Results were expressed as mean ± standard deviation (SD).

The anti-proliferative activity of the Se and Se@Ch–PTX NPs were evaluated in vitro on the MDA-MB-231 triple-negative breast cancer cell line using the MTT assay (Figure 5). The cell viability decreased with increasing concentrations of the Se@Ch–PTX NPs showing superior activity over PTX and over the Se NPs (Figure 5a). The IC_50_ value for Se@Ch–PTX of 14.3 µg/mL (Figure 5b, Table 2) recorded after 24 h is markedly higher than that of the components PTX (36.2 µg/mL) and Se NPs (52.4 µg/mL). It is noteworthy, that PTX was applied in equivalent mg amounts as the NPs in these experiments, which leads to a far higher overall PTX-content for the pure PTX material compared with the Se@Ch–PTX NPs. Which means that the use of the Se@Ch-based release system is superior to the application of pure PTX. The efficient release from the Se@Ch–PTX NPs aligns to the morphological changes observed in microcopy (Figure S6). Our findings agree very well with those of the quite similar Se NP-based PTX-release system Se@β-CD–FA@PTX (FA = folate, β-CD = β-cyclodectrin) [27], which showed markedly improved antiproliferative activity over the Se@β-CD–FA component or the Se NPs prepared from Na_2_SeO_3_ and ascorbic acid. The same has previously been reported for C-peptide-conjugated solid lipid NPs with an IC_50_ value of 1.0 µg/mL (Table 2) [67]. Our system is less active than this system, but can easily compete with other Se-based NP delivery systems (Table 2). Generally, Table 2 demonstrates that suitable coating of the Se NPs before loading them with PTX or co-loading other organic material improves the release and cytotoxicity of the NP materials.

Our findings and those of others (Table 2) also reveal that unloaded Se NPs show some antiproliferative activities. Presumably, the Se NPs contribute to the generation of reactive oxygen species (ROS), which play a key role in cytotoxic activity by overcoming the antioxidant defense mechanisms in tumor cells [24,27,28,29,58,69,70]. In our case, the activity might as well be enhanced by flavonoid and phenolic compounds present on the surface of the Se NPs originating from the *F. vulgare* seed extract. Therefore, the lower Se-content of the Se NPs compared with the Se@Ch–PTX (we used equivalent mg amounts) will probably not play a significant role. Our assumption aligns well with use of certain plant extracts leads to the formation of small-sized Se NPs, significantly enhancing cellular toxicity [33,34,42]. As many reported Se NPs for drug-delivery were synthesized using plant extracts or other biomaterials as reductants, the possibility that the biomaterials are far from being just “innocent” reductants requires a comparative study using drug-loaded Se NP produced using different biomaterials. We are planning such a study for the future.

### 2.7. Antioxidant Activities

**Figure 6:**
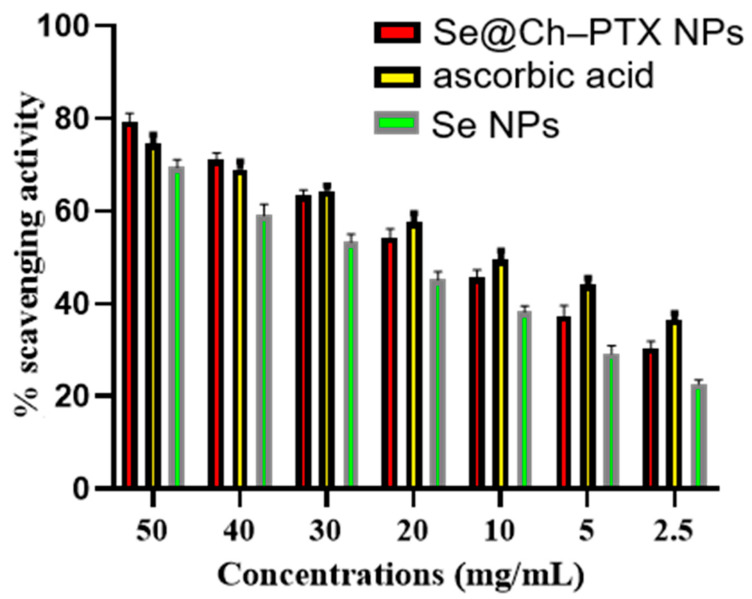
Antioxidant activities of Se NPs, Se@Ch–PTX NPs, and ascorbic acid in DPPH radical scavenging. All experiments were performed in triplicate. Results were expressed as mean ± standard deviation (SD).

The antioxidant potential of the Se NPs and the Se@Ch–PTX NPs was evaluated through a DPPH assay, using ascorbic acid as reference compound. All three samples showed concentration-dependent scavenging of DPPH free radicals (Figure 6). At the highest concentration of 50 mg/mL, ascorbic acid showed the highest activity with about 81% followed by the Se@Ch–PTX with 76% and the Se NPs with 71% (Table 3). Although ascorbic acid showed superior activity, the NPs demonstrated promising antioxidant potential, particularly Se@Ch–PTX, which consistently outperformed the Se NPs across all tested concentrations. This enhanced performance is further supported by the IC_50_ values, with the Se@Ch–PTX NPs showing higher activity (34 mg/mL) than the Se NPs (39 mg/mL), though being still less active than ascorbic acid (22 mg/mL) (Table 3).

Our values agree largely with those of similar materials in previous reports [25,27,42,57,69].

Overall, the chitosan-coating of our Se@Ch–PTX NPs seems to play a pivotal role in drug loading and release of PTX. This is probably due to the presence of amino and hydroxyl functional groups, which facilitate the encapsulation of PTX via electrostatic and hydrophobic interactions. These interactions not only improve drug loading efficiency but might also contribute to a sustained release profile as has been pointed out before [59,60,62,63,64]. Collectively, the multifunctional role of chitosan includes nanoparticle stabilization, enhanced drug-loading capacity, and favorable surface characteristics that improve drug release. Potentially, chitosan also improves cellular uptake, but we have not studied this point herein.

## 3. Materials and Methods

### 3.1. Materials

Sodium selenite Na_2_SeO_3_ (>90%RT, Sigma Aldrich, Merck, Darmstadt, Germany), Paclitaxel (PTX, C_47_H_51_NO_14_, M_W_ = 853.91 g/mol; Merck, Darmstadt, Germany), chitosan ((C_6_H_11_O_4_N)_n_, 684 kDa, deacetylation approx. 84%, Merck, Darmstadt Germany), Triton-X-100 (Sigma Aldrich), and dimethyl sulfoxide (DMSO, C_2_H_6_SO, Merck) were used as purchased. *F. vulgare* seeds were purchased from local markets in the city of Habbaniyah, Iraq. The seeds were carefully dry-cleaned, washed with distilled water, and carefully dried.

### 3.2. Preparation of F. vulgare Seed Extract

In accordance with a previous report [54,70], 100 g of the *F. vulgare* seeds were ground into a fine powder using an electric grinder (Moulinex AR1100, Groupe SEB, Écully, France), mixed with 250 mL of distilled water, and heated to 45 °C for 4 h. The mixture was left to cool to room temperature and filtered using a Whatman filter paper No. 1.

### 3.3. Synthesis of Se NPs

Adopting reported procedures [35,36], we initially tested different volumes of *F. vulgare* seed extract for the NP production (1, 2, 3, and 5 mL) and monitored the speed of the reaction by UV–vis absorption and the completeness of the reaction by analyzing the yields. The optimized conditions were 2 mL of freshly prepared *F. vulgare* seed extract added slowly to 2 g (11.5 mmol) of sodium selenite dissolved in 15 mL H_2_O. The mixture was stirred thoroughly for 60 min at 30 °C, followed by centrifugation for 15 min at 10,000 rpm. The NPs were washed with distilled water and then dried at 50 °C, resulting in 810 mg (10 mmol, 87%) red Se NP material. The NPs immediately were suspended in a phosphate-buffered saline (PBS) solution (pH = 7.4) using ultrasound and directly used for coating and loading.

### 3.4. Preparation of the Chitosan-Coated Se NPs—Se@Ch

In initial experiments, we prepared chitosan solutions in glacial acetic acid with varying contents (0.2%, 0.5%, 1%, and 2% *v*/*v*) and studied the NP stability and Zeta potentials. The optimum condition was a 1% solution, stirred for 90 min to ensure complete homogeneity. Then, 1 g of Se NPs were added with continuous stirring at room temperature for 10 h. After centrifugation, the supernatant was discarded and the NP precipitate washed twice with water.

### 3.5. Loading with Paclitaxel (PTX)

In initial loading experiments were prepared solutions of different concentrations of PTX in DMSO (10 mg, 25 mg, and 40 mg in 5 mL DMSO) to find the optimum conditions. The speed and the efficiency of the loading was tested through the PTX content in the supernatant solutions using HPLC. The optimum conditions were 25 mg Paclitaxel (PTX) dissolved in 5 mL DMSO and added dropwise at a rate of 1 mL/min to the Se@Ch NPs. The mixture was left stirring for 16 h, then the solution was centrifuged at 10,000 rpm for 20 min. The supernatant was discarded and the resulting precipitate was washed three times with 5 mL of distilled water and finally air-dried. The last supernatant was free from PTX as confirmed through HPLC. The Se@Ch–PTX samples were stored at room temperature for further analysis and characterization.

### 3.6. Instrumentation

Field emission scanning electron microscopy (FESEM) was carried out using a FESEM MIRA3 TESCAN-XMU (Tescan, Brno, Czech Republic, set at 20 kV. Transmission electron microscopy (TEM) images were obtained using a JEM-1230 instrument (JEOL, Akishima, Tokyo, Japan) with an acceleration voltage of 200 kV. Powder X-ray diffraction was performed using a Bruker AXSD8 instrument (D8 FOCUS 2200 V Bruker AXS, Rheinhausen, Germany), employing Cu Kα radiation with a 2θ range from 20° to 80°. Diffuse reflectance spectra (DRS) at a resolution of 4 cm^−1^ were recorded using Perkin Elmer 1750 FTIR spectrophotometer (PerkinElmer, Waltham, MA, USA). The Zeta potential was measured using a Malvern Zetasizer Nano (Malvern Panalytical, Malvern, UK). For sample preparation, the Se@Ch–PTX NPs were dispersed in distilled water at a concentration of 4 mg/mL and sonicated for 15 min. Then, approximately 900 µL of the dispersion was injected into the DTS1070 cells, and Zeta potential measurements were conducted at 25 °C. The collected data were analyzed using Zetasizer Software version 8.02. Thermogravimetric Analysis (TGA) was performed using a SETSYS Evolution TGA-DTA/DSC instrument (Setaram Instrumentation, Caluire-et-Cuire, France) on powdered samples.

### 3.7. In Vitro PTX Release

A suspension of 10 mg of the NPs was prepared in 10 mL of buffer solution for each pH = 3.5, 7.4, and 9.0. The mixture was transferred to dialysis tubes (SERVA Electrophoresis GmbH, Heidelberg, Germany) with a pore size of 25 inches (M_W_CO = 12,000–14,000 Da). The dialysis tubes were immersed in 50 mL of the corresponding buffer solution in a water bath maintained at 37 °C, with continuous stirring at 100 rpm. Samples of 1 mL were taken at 0, 1, 2, 4, 6, 8, 12, 18, and 24 h. After each sample collection, the same volume of fresh buffer solution was added to maintain the volume. The PTX concentration in the collected samples was analyzed using an Agilent 1260 Infinity II High-Performance Liquid Chromatography (HPLC) (Agilent, Santa Clara, CA, USA). A C18 column (250 mm × 4.6 mm; 5 μm) was employed. Detection was carried out at 227 nm. The mobile phase consisted of an 80:20 (*v*/*v*) mixture of MeOH and water, the flow rate was 1 mL/min, ensuring efficient separation of the sample components [71]. A 20 μL volume of each sample was injected into the system using an auto-injector. The drug release percentage was calculated by comparing the concentration of PTX in the analyzed samples to the total amount of drug loaded onto the NPs (determined using HPLC). All measurements were conducted in triplicate.

### 3.8. Hemolytic Activity Assay

The hemolytic activity was evaluated using a previously reported method [72]. A fresh human blood sample was collected from three healthy donors (age 24–30 years) and prepared by centrifugation at 800× *g* for 10 min, followed by three washes with normal saline. The red blood cells (RBCs) were suspended in normal saline (10% *v*/*v*), and 200 μL of this suspension was mixed with 200 μL of each concentration of the NPs (25, 50, 100, 200, and 400 μg/mL). The mixture was incubated at 37 °C for 60 min. Then, the samples were centrifuged at 10,000 rpm for 5 min. A total of 100 μL of each sample was transferred to a 96-well plate to measure absorbance at 540 nm using an Agilent BioTek Synergy H1 microplate reader (Agilent, Santa Clara, CA, USA). Triton-X-100 and normal saline were used as positive and negative controls, respectively. The hemolytic activity of the NPs was calculated using Equation (1).


%Hemolysis = (absorbance of NPs − absorbance of negative control)/(absorbance of positive control − absorbance of negative control) × 100
(1)

### 3.9. Coagulation Time Assay

The effect of Se@Ch–PTX NPs on coagulation time was assessed through aPTT (activated partial thromboplastin time) and PT (prothrombin time) assays [73]. Briefly, fresh blood samples were collected from healthy donors (aged 25–30 years) and centrifuged at 2500× *g* for 15 min to obtain platelet-poor plasma. A total of 100 µL of Se@Ch–PTX NPs at concentrations of 50, 100, 200, and 400 µg/mL was dispersed in 900 µL of the platelet-poor plasma and incubated for 30 min at 37 °C. Then, the suspension of each sample was centrifuged at 18,000× *g* for 5 min for both assays. Coagulation time was measured using the Sysmex CA-7000 analyzer (Sysmex Corporation, Kobe, Japan) for both aPTT and PT tests. The results were presented as mean ± standard error and compared to the control samples (normal saline).

### 3.10. Antiproliferative Activity

The MDA-MB-231 human breast cancer cell line was obtained from the Pasteur Institute of Tunis. Cells were cultured in Dulbecco’s Modified Eagle Medium (DMEM) supplemented with 10% fetal bovine serum (FBS) and 1% antibiotic–antimycotic solution. Cell cultures were maintained under standard incubation conditions at 37 °C in a humidified atmosphere containing 5% CO_2_, with routine sub-culturing to ensure viability and genetic stability. in Dulbecco’s Modified Eagle Medium (DMEM) supplemented with 10% fetal bovine serum (FBS) and 1% antibiotic–antimycotic solution. Cell cultures were maintained under standard incubation conditions at 37 °C in a humidified atmosphere containing 5% CO_2_, with routine sub-culturing to ensure viability and genetic stability. Once the cells reached approximately 70–80% confluency, they were harvested for use in cytotoxicity assays. Cells were seeded into 96-well microplates at a density of 1 × 10^5^ cells per well and incubated for 24 h to allow for proper attachment [74,75]. Subsequently, the cells were treated with serial concentrations of Se@Ch–PTX NPs, Se NPs, and PTX ranging from 3.12 to 200 µg/mL, followed by 72 h incubation under identical conditions. To assess the antiproliferative activity, a standard MTT assay was employed. Following treatment, the medium was carefully removed, and 25 µL of MTT solution (5.5 mg/mL) was added to each well. Plates were wrapped in aluminum foil to protect from light and incubated for an additional 4 h. Subsequent to incubation, the MTT reagent was aspirated, and 100 µL of dimethyl sulfoxide (DMSO) was added to each well to solubilize the resulting formazan crystals. Absorbance was measured at 570 nm and 650 nm using a UV–vis microplate reader (BioTek Synergy H1, Agilent Technologies, Santa Clara, CA, USA). Untreated wells were designated as the negative control group for reference. Cell viability (%) was calculated using Equation (2):


Cell viability (%) = [(OD_570_ − OD_650_)_test ÷ (OD_570_ − OD_650_)_control] × 100 (2)

The half maximal inhibitory concentration (IC_50_), representing the concentration required to inhibit 50% of cell growth, was determined using Equation (3):


Inhibition (%) = [(OD_control − OD_test) × 100] ÷ OD_control
(3)

All treatments were performed in triplicate. Results were expressed as mean ± standard deviation (SD). Statistical analysis was carried out using SPSS version 25.0 (IBM Corp., Armonk, NY, USA), and differences were considered statistically significant at *p* < 0.05. The cell morphology of MDA-MB-231 cells was studied using a CKX53 phase-contrast light microscope (Olympus, Tokyo, Japan).

### 3.11. Antioxidant Activity Assay Using DPPH

The colorimetric method based on the compound DPPH (1,1-diphenyl-2-picrylhydrazyl) was employed to evaluate the antioxidant activity NPs, following a previously reported protocol [76]. The samples were prepared through serial dilutions, in which equal volumes of the sample and MeOH were mixed to obtain the following concentrations: 5, 10, 20, 30, 40, and 50 mg/L. Then, 1 mL of DPPH solution (*c* = 0.135 mM) was added to each sample. Ascorbic acid was used as a reference. After incubating the samples in the dark at 298 K for 30 min, the absorbance was measured at a wavelength of 517 nm using a UV–vis absorption spectrophotometer (UV-2600i, Shimadzu, Japan). The percentage of free radical scavenging (antioxidant activity) was calculated using Equation (4), with the DPPH solution in MeOH serving as the control.


% Radical Scavenging = ((A_control − A_sample)/A_control) × 100
(4)

This procedure was based on previously published methods with only minor modifications [77]. To determine the half-maximal inhibitory concentration (IC_50_), an exponential curve was used to illustrate the relationship between sample concentration and the remaining percentage of DPPH• free radicals [78].

### 3.12. Statistical Analysis

Data are presented as mean ± standard error (SE). A one-way analysis of variance (ANOVA) was employed to assess differences between group means. A *p*-value of less than 0.05 was considered to indicate statistical significance.

## 4. Conclusions

Selenium nanoparticles (Se NPs) were synthesized from Na_2_SeO_3_ using *Foeniculum vulgare* (fennel) seed extract as mild sustainable reductant, coated with chitosan (Ch), and loaded with Paclitaxel (PTX). The PTX release from the Se@Ch–PTX NPs and the cytotoxicity against MDA-MB-231 cells was studied in view of an application as drug delivery platform for the treatment of breast cancer. Thermogravimetric Analysis (TGA) showed thermal stability of the NPs up to 300 °C, the UV–vis absorption and Fourier-transform IR spectroscopy allowed to trace surface species originating from the *F. vulgare* extract on the Se NP, while the surface of the Se@Ch–PTX NPs seems to be dominated by functionalities originating from Ch and PTX. This agrees with the positive Zeta potential of +57 mV for the Ch/PTX coated NPs in contrast to the −39 mV found the Se NPs. The NPs showed good biocompatibility with red blood cells in hemolytic activity assays, exhibiting no hemolytic effects at concentrations ranging from 50 to 400 µg/mL. In vitro release studies indicated a sustained and pH-responsive release pattern with a maximum release of about 80% within 22 h for Se@Ch–PTX at pH = 3.5, which is perfect for medical application. The Se@Ch–PTX NPs showed high antiproliferative activity against MDA-MB-231 with an IC_50_ value of 12.3 µg/ML compared to about 38 for PTX and 55 for the Se NPs. The reactive oxygen species (ROS) activity as studied through DPPH scavenging showed higher values for the Se@Ch–PTX NPs compared to the Se NP. The modeling of the release kinetics showed high conformity with the Korsmeyer–Peppas model, which is characteristic of drug release through a combined mechanism involving both the diffusion and degradation of the biopolymer coating. Thus, the synthesis of the Se NPs using *F. vulgare* extract is a reliable and robust method. The NPs were coated with chitosan and this cover impregnated with PTX. Compared with other Se NP-based drug delivery systems for PTX, Se@Ch–PTX is superior in its stability and controlled release. The high antiproliferative activity of Se@Ch–PTX is mainly due to the PTX release. However, comparison with other work showed that the Se NP carrier also contributed through production of ROS. Our study also illustrates the beneficial role of chitosan in nanoparticle stabilization, drug-loading capacity, and surface characteristics that are likely to improve drug-release. Furthermore, when using biomaterials as reductants for the production of such Se NPs, remainders of these materials were found as surface-protecting ligands on the NPs and are probably not entirely removed during subsequent processes such as coating or drug-loading. Thus, these ligands/species might also contribute to the biological activities of such NPs and could thus be selected on purpose. Unfortunately, there are not enough reports so far to evaluate this interesting approach. But this might be an interesting area for future work.

## Figures and Tables

**Table 1:** Release rate constant (*K*) and regression coefficient (*R*^2^) for different release models ^a^.

pH	Zero-Order		First-Order		Higuchi		Korsmeyer-Peppas	
	*K*_0_	*R* ^2^	*K*_1_	*R* ^2^	*K*_H_	*R* ^2^	*K*	*R* ^2^
9.0	1.85	0.90	0.0164	0.8118	0.9835	0.9835	0.3835	0.9905
7.4	1.54	0.89	0.0163	0.8038	0.9802	0.9802	0.3825	0.9887
3.5	2.21	0.85	0.0176	0.7393	0.9618	0.9618	0.4287	0.9695

^a^ For the release of PTX from Se@Ch–PTX NPs (see Figure 3).

**Table 2:** Comparison of reported IC_50_ values of Se NP formulations against cancer cells ^a^.

Delivery System ^b^ [Incubation]	IC_50_ (µg/mL) [Cell Line] ^c^	Reference
Se@Ch–PTX NPs [24 h]	14.3 [MDA-MB-231]	this work
PTX/Se NPs [24]	36.2/52.4 [MDA-MB-231]	this work
PTX@Se NPs ^b^ [48 h or 72 h]	2.98 [MCF-7], 0.79 [HeLa]	[25]
Se@Ch–PTX NPs [24 h]	30.0 [HeLa]	[26]
Se@HA–PTX ^d^ [24 h]	4 [A549]	[28]
Se@PTX [24 h]	8 [A549]	[28]
Se@PTX ^e^ [48 h]	13.8 [A549], 5.4 [MCF-7], 8.7 [HeLa], 4.8 [HT-29]	[29]
Se NPs [48 h]	28.1 [A549], 12.2 [MCF-7], 25.3 [HeLa], 10.9 [HT-29]	[29]
C-peptide–SLN–PTX ^f^ [24 h]	1.2 [4T1] ^e^, 1.0 [MDA-MB-231]	[67]
SLN–PTX [24 h]	3.4 [4T1], 4.0 [MDA-MB-231]	[67]
C-peptide/PTX [24 h]	10.7/8.9 [4T1] 9.8/8.3 [MDA-MB-231]	[67]

^a^ In each case an MTT assay was used. ^b^ Diselenide-containing PTX NPs. ^c^ MCF-7 is a breast cancer cell line, 4T1 is a triple-negative breast cancer cell line, HeLa is a cervical cancer cell line, HT-29 is a colon cancer cell line. ^d^ Prepared from Na_2_SeO_3_, ascorbic acid, hyaluronic acid (HA). ^e^ Prepared from SeO_2_, ascorbic acid, pluronic F-127. ^f^ C-peptide-conjugated solid lipid nanoparticles, prepared from stearic acid, Precirol, Tween 20, lecithin, and Poloxamer 407.

**Table 3:** DPPH-scavenging activity (%) and IC_50_ (mg/mL) values ^a^.

Concentrations (mg/mL)	Ascorbic Acid	Se@Ch–PTX NPs	Se NPs
50	80.64 ± 1.56	75.91 ± 1.27	70.63 ± 1.36
40	72.12 ± 1.37	70.25 ± 0.89	60.78 ± 1.32
30	64.22 ± 1.91	65.15 ± 0.71	54.52 ± 1.28
20	55.64 ± 1.35	58.96 ± 0.56	46.42 ± 0.98
10	46.81 ± 1.28	50.85 ± 0.47	39.12 ± 0.54
5	38.94 ± 1.08	45.12 ± 0.48	30.36 ± 0.28
2.5	31.47 ± 1.12	37.56 ± 0.51	23.21 ± 0.38
IC_50_ (mg/mL) ^b^	22.03	33.72	39.44
*F* value ^c^	1.91 ***	1.52 ***	1.63 ***

^a^ Values significance at probability level of 0.001 (*** *p* < 0.001). ^b^ IC_50_ = half-maximal inhibitory concentration. ^c^ *F* value = ratio between the variance caused by differences between groups (between-group variance) and the variance caused by differences within each group (within-group variance).

## Data Availability

The data presented in this study are available on request from the corresponding authors.

## Supplementary Materials

The following supporting information can be downloaded at: https://www.mdpi.com/article/10.3390/nano15161276/s1. The materials contain Figure S1: FT-IR spectra of PTX, Se NPs, *F. vulgare* seed extract, and Se@Ch–PTX NPs. Figure S2: UV–vis absorption spectra of *F. vulgare* seed extract, Se NPs, and Se@Ch-PTX NPs. Figure S3: Powder X-ray diffractograms of Se NPs, Se@Ch-PTX NPs, and chitosan with assigned reflexes for Se (hexagonal; JCPDS file number 06-0362). Figure S4: TGA of the Se@Ch-PTX NPs. Figure S5: Zeta potential measurements of (a) Se NPs and (b) Se@Ch-PTX NPs. Figure S6: Morphological changes in MDA-MB-231 breast cancer cells after treatment with a concentration of 200 mg/mL for 72 h using PTX, Se NPs, and the Se@Ch–PTX NPs.
