# Supplementary material for: Green Synthesis of Chitosan-Coated Selenium Nanoparticles for Paclitaxel Delivery
Source: Nanomaterials (Basel). 2025 Aug 18;15(16):1276. doi: 10.3390/nano15161276 (PMC12388513; doi:10.3390/nano15161276)
Supplement: Supplementary file 1 [file nanomaterials-15-01276-s001.zip › nanomaterials-3754662-supplementary.pdf]

Supplementary Materials
for
Green Synthesis of Chitosan-Coated Selenium Nanoparticles for Paclitaxel Delivery

Mouhaned Y. Al-Darwesh [1,2,*], Maroua Manai [3], Hammouda Chebbi [1,4], and Axel Klein [5,*]

[1] University of Tunis El Manar, Faculty of Sciences of Tunis, Laboratory of Materials, Crystal Chemistry and Applied Thermodynamics, 2092 El Manar II, Tunis, Tunisia. Email: chebhamouda@yahoo.fr, ORCID: 0000-0002-0726-7053 (H.C.)
[2] Department of Chemistry, College of Science, University of Anbar, Ramadi, Iraq,
[3] Laboratory of Transmission, Control and Immunobiology of Infections, Pasteur Institute of Tunis, Tunis, Tunisia. Email: marwa.manai@pasteur.utm.tn , ORCID: (0000-0003-4037-3402.)
[4] University of Tunis, Preparatory Institute for Engineering Studies of Tunis, Street Jawaharlal Nehru, 1089, Montfleury, Tunis, Tunisia.
[5] Institute for Inorganic and Materials Chemistry, Department of Chemistry and Biochemistry, Faculty of Mathematics and Natural Sciences, University of Cologne, Greinstrasse 6, 50939 Köln, Germany.

* Corresponding authors: Mouhaned Y. Al-Darwesh (Email: myturky@uoanbar.edu.iq); ORCID: 0000-0003-1153-1408 (M.D.); Axel Klein, Email: axel.klein@uni-koeln.de, ORCID: 0000-0003-0093-9619 (A.K.)

Contents

Figure S1. FT-IR spectra of PTX, Se NPs, *F. vulgare* seed extract, and Se@Ch–PTX NPs.
Figure S2. UV-vis absorption spectra of *F. vulgare* seed extract, Se NPs and Se@Ch-PTX NPs.
Figure. S3. Powder X-ray diffractograms of Se NPs, Se@Ch-PTX NPs, and chitosan with assigned reflexes for Se (hexagonal; JCPDS file number 06-0362).
Figure S4. TGA analysis of the Se@Ch-PTX NPs.
Figure S5. Zeta potential measurements of (a) Se NPs and (b) Se@Ch-PTX NPs.
Figure S6. Morphological changes in MDA-MB-231 breast cancer cells after treatment with a concentration of 200 mg/mL for 72 h using PTX, Se NPs, and the Se@Ch–PTX NPs. Black arrows indicate floating dead cells.

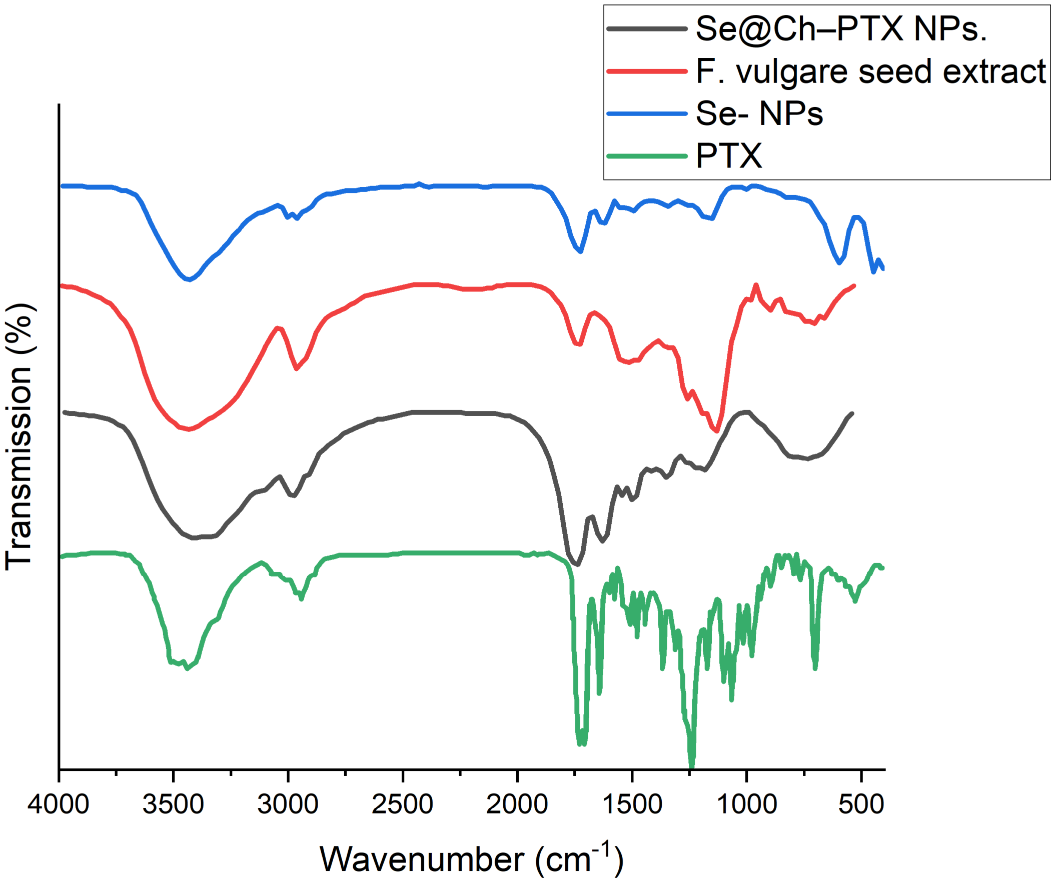

Figure S1. FT-IR spectra of PTX, Se NPs, *F. vulgare* seed extract, and Se@Ch–PTX NPs.

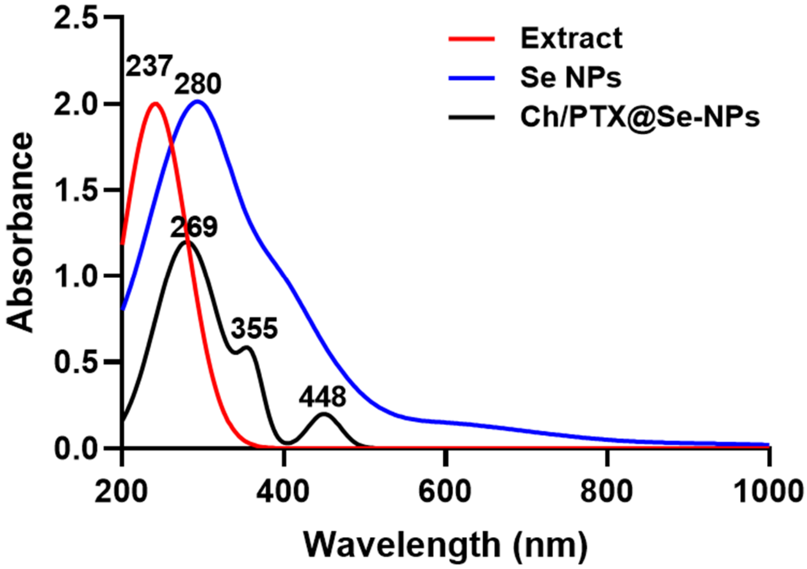

Figure S2. UV-vis absorption spectra of *F. vulgare* seed extract, Se NPs and Se@Ch–PTX NPs.

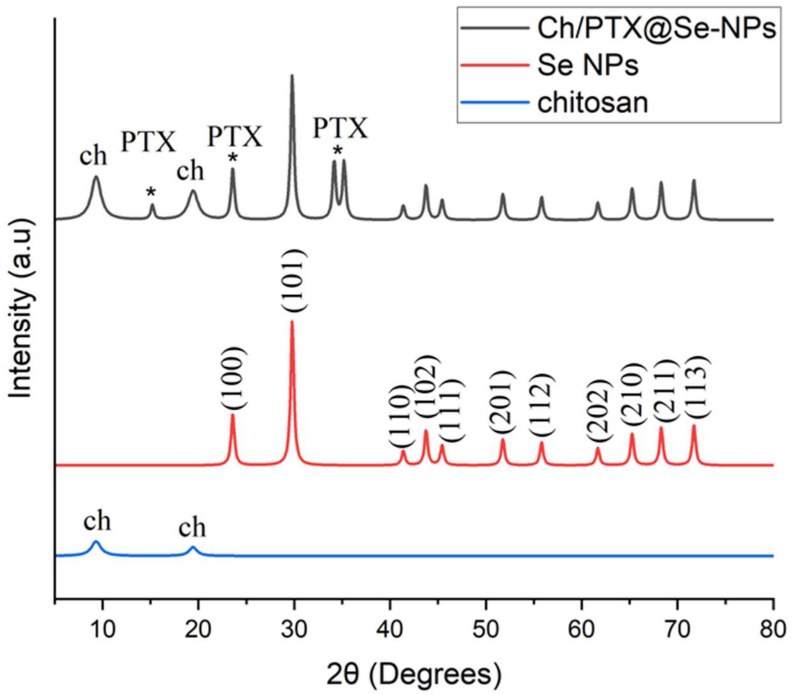

Figure. S3. Powder X-ray diffractograms of Se NPs, Se@Ch–PTX NPs, and chitosan with assigned reflexes for Se (hexagonal; JCPDS file number 06-0362).

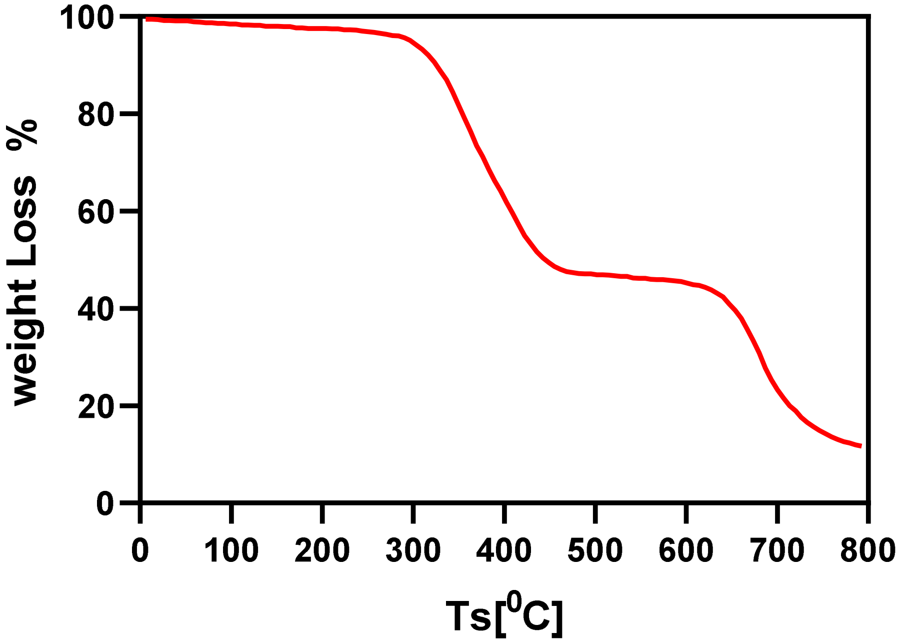

Figure S4. TGA analysis of the Se@Ch–PTX NPs.

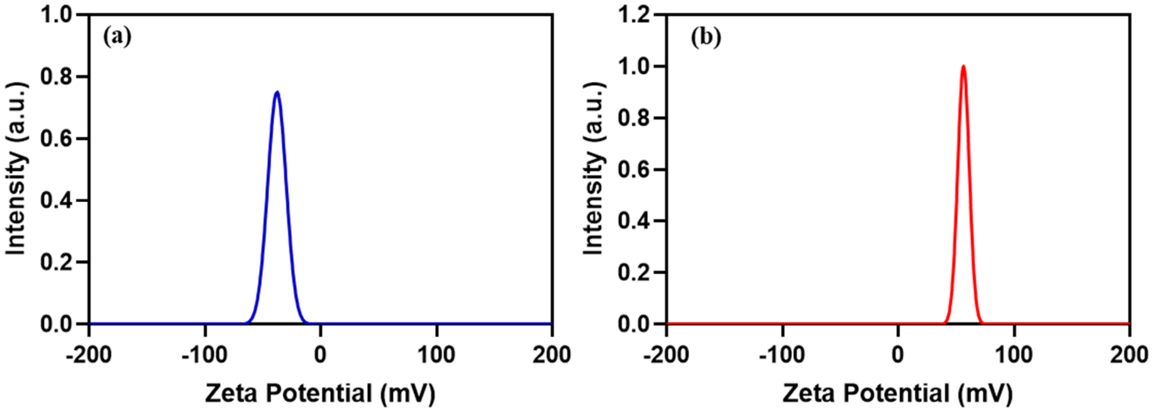

Figure S5. Zeta potential measurements of (a) Se NPs and (b) Se@Ch–PTX NPs.

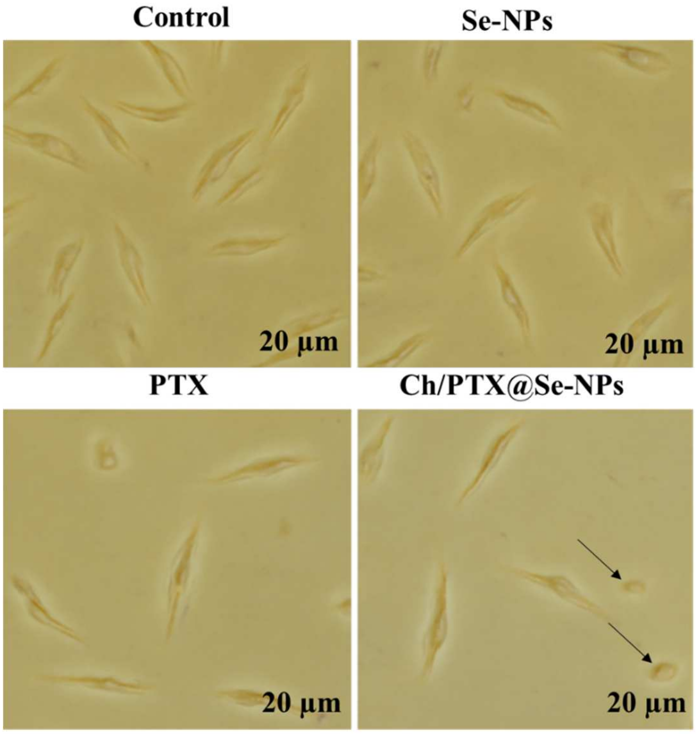

Figure S6. Morphological changes in MDA-MB-231 breast cancer cells after treatment with a concentration of 200 mg/mL for 72 h using PTX, Se NPs, and the Se@Ch–PTX NPs. Black arrows indicate floating dead cells.
